# Musculoskeletal disorders in women of childbearing age: global trends, socio-demographic disparities, and future projections

**DOI:** 10.1080/07853890.2025.2532860

**Published:** 2025-07-20

**Authors:** Liang Luo, Xīn Gào, Junting Yang, Xuewu Zhang, Zhike Liu, Chun Li

**Affiliations:** a Department of Chinese Medicine, The People’s Hospital of Yubei District of Chongqing, Chongqing, China; b Department of Rheumatology and Immunology, Peking University People’s Hospital, Beijing, China; c Department of Epidemiology and Biostatistics, School of Public Health, Peking University, Beijing, China; d Key Laboratory of Epidemiology of Major Diseases, Peking University, Ministry of Education, Beijing, China

**Keywords:** Disease burden, musculoskeletal disorders, women of childbearing age, projection, Socio-Demographic Index

## Abstract

**Background:**

Musculoskeletal (MSK) disorders are a leading cause of disability worldwide, particularly prevalent among women of childbearing age (WCBA). Our aim is to comprehensively assess the global, regional, and national burden of MSK disorders in WCBA, and examine the burden of MSK disorders among WCBA at varying levels of the Socio-demographic Index (SDI), then to project the burden of these disorders through to 2045.

**Methods:**

This study utilized data from the Global Burden of Disease (GBD) 2021 project, focusing on MSK disorders among WCBA (15–49 years). Age-standardized rate was calculated using the World Standard Population proportions. Descriptive analysis was conducted at global, regional, and national levels. SDI associations were explored using smoothing spline models. Projections to 2045 employed age-period-cohort models using R software.

**Results:**

In 2021, the estimated global age-standardized incidence, prevalence, deaths, Years of Life Lost, Years Lived with Disability, and Disability-Adjusted Life Years (DALYs) rates per 100 000 population of MSK disorders in WCBA were 4933.9 (95% UI 3683.7–6454.4), 20145.9 (17 082.6–23 564.4), 0.7 (0.6–0.8), 40.7 (34.1–45.7), 2090.4 (1414.8–2896.2), and 2131.1 (1455.8–2936.8), respectively. From 1990 to 2021, a total of 183 countries exhibited an increase in prevalence rate and 166 countries showed an upward trend in DALYs rate. Between 1990 and 2021, there was a positive association between the SDI and age-standardized DALYs rate for MSK disorders in WCBA, both globally and regionally. By 2045, the age-standardized number of DALYs for MSK disorders in WCBA is expected to reach 48.8 million, with an age-standardized DALYs rate of 2160 per 100,000 population.

**Conclusion:**

The burden of MSK disorders among WCBA is already substantial and is expected to increase further in the future. Despite the observed decline in age-standardized incidence rate of MSK disorders among WCBA in half of the regions and countries globally, the age-standardized prevalence and DALYs rates have shown an adverse increasing trend. By 2045, the global number of DALYs for MSK disorders in WCBA is projected to exceed 48 million. To mitigate the future burden of MSK disorders in WCBA, stratified and targeted healthcare strategies are essential to improve early diagnosis and treatment.

## Introduction

Musculoskeletal (MSK) disorders encompass a range of conditions affecting the MSK system, including the joints, bones, tendons, muscles, ligaments, and spine [1] and are a leading cause of disability worldwide [2]. However, the significance of musculoskeletal disorders is often underestimated because they are rarely fatal [3]. Despite their lower mortality rate, these conditions can cause significant long-term disability and diminished quality of life. They frequently lead to chronic pain, impaired mobility, and reduced functional capacity, which can severely impact daily activities and overall well-being [4].

MSK disorders are more prevalent in women, particularly during their childbearing age [5,6]. This population is uniquely vulnerable to MSK disorders due to hormonal fluctuations, pregnancy-associated stressors, and socioeconomic challenges impacting reproductive health. In 2019, the global number of women of childbearing age (WCBA) with MSK disorders was 354.57 million [7]. According to the 2024 World Population Prospects report, the global number of WCBA is projected to increase from nearly 2 billion in 2024 to approximately 2.2 billion by the end of the 2050s [8]. The rising burden of MSK disorders in WCBA has emerged as a key driver of declining fertility rates [9], acting through direct physiological disruptions to reproductive health and indirect intensification of socioeconomic challenges. This trend is expected to intensify population aging and increase societal and economic burdens. Although previous studies have reported on the global burden of MSK disorders or specific MSK conditions [10–14], there is a lack of research specifically addressing the burden of these diseases in WCBA. Robust evidence on the impact of regional differences and socio-demographic development on the burden of MSK disorders in WCBA remains limited.

The primary aim of this study is to comprehensively assess the global, regional, and national burden of MSK disorders among WCBA. By analyzing data from 1990 to 2021, we investigate the trends in incidence, prevalence, and Disability-Adjusted Life Years (DALYs) rates of MSK disorders across different regions and countries worldwide. Additionally, we aim to examine whether there are significant differences in the burden of MSK disorders among WCBA at varying levels of the Socio-demographic Index (SDI) and to project the burden of these disorders through to 2045. Through this study, we hope to provide robust data and scientific evidence to inform the control and prevention of MSK disorders in WCBA at global, regional, and national levels, thereby improving the health status of this population.

## Methods

### Data source

This study utilized data from the Global Burden of Disease (GBD) 2021 project, which provides comprehensive estimates of the epidemiological burden of 371 diseases and injuries across 21 GBD regions and 204 countries and territories from 1990 to 2021 [15]. Specifically, we focused on MSK disorders, including rheumatoid arthritis (RA), osteoarthritis (OA), low back pain (LBP), neck pain (NP), gout, and other musculoskeletal disorders (OMSKD), among WCBA (the age group of 15 to 49 years). The data on incidence, prevalence, deaths, Years of Life Lost (YLLs), Years Lived with Disability (YLDs), and DALYs are publicly accessible *via* the Global Health Data Exchange (GHDx) and the GBD Compare tool provided by the Institute for Health Metrics and Evaluation (IHME). Detailed methodological information and statistical modeling approaches are documented in previous GBD publications [3]. For comprehensive insights, refer to the GBD Results Tool at http://ghdx.healthdata.org/gbd-results-tool and the GBD Compare tool at https://vizhub.healthdata.org/gbd-compare/

### Case definition

The GBD classification of MSK comprises six major categories, including RA, OA, LBP, NP, gout, and OMSKD. Detailed definitions and corresponding ICD codes for these diseases are provided in Supplementary Table 1.

According to the World Health Organization, WCBA are defined as those aged from 15 to 49 years. This demographic is characterized by high fertility and cyclical changes in sex hormones [16].

### Data processing and disease modeling

To comprehensively understand the burden of MSK disorders in WCBA, a descriptive analysis was conducted at global, regional, and national levels. In this study, estimates and 95% uncertainty intervals (UIs) for the number of MSK disorders cases were extracted from GBD 2021 at the global, regional (5 SDI regions and 21 GBD regions), and national (204 countries and territories) levels, across seven age groups (15–19, 20–24, 25–29, 30–34, 35–39, 40–44, and 45–49 years). Age-standardization of incidence, prevalence, deaths, YLLs, YLDs, and DALYs for MSK disorders in WCBA was conducted by weighting the age-specific rate by the corresponding proportions of the World Standard Population for each age group, resulting in standardized data. The GBD standard Cause of Death Ensemble model (CODEm) was used to estimate mortality due to MSK disorders in WCBA. The estimates for each metric include 95% UIs, derived from the 2.5th and 97.5th percentiles among 1,000 estimates according to the GBD algorithm. All rates are presented per 100,000 population.

The Socio-Demographic Index (SDI) consists of three components: lag-distributed income per capita (GDP per capita averaged over the previous 10 years), mean education level for individuals aged 15 and older, and the total fertility rate for women under 25. The SDI ranges from 0 (least developed) to 1 (most developed). Smoothing spline models were utilized to explore the associations between the DALYs rate of MSK disorders in WCBA and the SDI across 21 regions [17].

### Estimate projections

A linear log age-time-cohort model was utilized to forecast the number and age-standardized rates of incidence, prevalence, and DALYs from 2022 to 2045. Exponential growth trends were effectively managed, and linear trend projections were constrained by this model. The setup and parameter estimation were executed using the Nordpred package in R [18]. Generalized linear models were employed by Nordpred to account for age, period, and cohort effects, enabling robust predictions of future disease burden.

Data cleaning, computation, and graph plotting for this study were performed using R software (version 4.4.1). Visualizations were generated with the ggplot2 package.

## Result

In 2021, the estimated global age-standardized incidence, prevalence, deaths, YLLs, YLDs, and DALYs rates per 100,000 population of MSK disorders in WCBA were 4933.9 (95% UI 3683.7–6454.4), 20 145.9 (17 082.6–23 564.4), 0.7 (0.6–0.8), 40.7 (34.1–45.7), 2090.4 (1414.8–2896.2), and 2131.1 (1455.8–2936.8), respectively (Table 1). At the regional level, High-income North America had the highest age-standardized prevalence, YLDs, and DALYs rates. Australasia, the Caribbean, and Central Latin America had the highest age-standardized incidence, deaths, and YLLs rates, respectively (Table 1).

**Table 1.** Age-standardised rates of incidence, prevalence, deaths, YLLs, YLDs and DALYs per 100,000 population caused by MSK disorders in women of childbearing age at global and region levels in 2021.

Location	Incidence	Prevalence	Deaths	YLLs	YLDs	DALYs
Global	4933.9 (3683.7, 6454.4)	20145.9 (17082.6, 23564.4)	0.7 (0.6, 0.8)	40.7 (34.1, 45.7)	2090.4 (1414.8, 2896.2)	2131.1 (1455.8, 2936.8)
Central Sub-Saharan Africa	4628.7 (3419.5, 6138.0)	16432.2 (13464.9, 19771.7)	0.8 (0.3, 1.5)	44.0 (18.8, 84.1)	1693.6 (1118.6, 2374.0)	1737.5 (1159.3, 2428.4)
Eastern Sub-Saharan Africa	4469.3 (3306.5, 5889.6)	15551.7 (12790.6, 18589.5)	0.4 (0.2, 0.7)	22.0 (13.0, 42.5)	1608.8 (1063.8, 2264.7)	1630.8 (1091.2, 2287.8)
Western Sub-Saharan Africa	4716.0 (3485.7, 6228.8)	17239.8 (14215.7, 20797.9)	0.9 (0.5, 1.2)	47.0 (25.3, 65.4)	1798.8 (1200.0, 2525.6)	1845.8 (1249.8, 2575.2)
Southern Sub-Saharan Africa	4237.2 (3132.6, 5580.9)	16272.0 (13484.5, 19365.0)	1.2 (0.9, 1.7)	65.9 (47.6, 88.0)	1640.6 (1099.7, 2273.2)	1706.5 (1167.0, 2341.9)
North Africa and Middle East	6107.3 (4487.0, 8062.8)	22929.8 (19006.3, 27455.0)	0.7 (0.5, 1.0)	42.5 (31.1, 55.4)	2466.7 (1643.8, 3428.9)	2509.2 (1686.4, 3472.0)
South Asia	4806.6 (3554.7, 6368.6)	22405.4 (18758.6, 26344.1)	0.3 (0.3, 0.4)	18.1 (13.7, 22.1)	2289.7 (1554.2, 3171.9)	2307.8 (1572.9, 3189.9)
Oceania	4164.7 (3084.9, 5447.7)	15660.1 (12970.0, 18673.9)	0.6 (0.2, 1.2)	39.6 (12.9, 74.2)	1583.4 (1047.2, 2225.5)	1623.0 (1087.6, 2266.8)
Southeast Asia	4117.2 (3049.1, 5440.0)	17169.3 (14406.3, 20232.9)	1.1 (0.9, 1.4)	66.9 (51.6, 82.4)	1771.4 (1185.6, 2472.9)	1838.3 (1252.7, 2537.9)
East Asia	3631.3 (2754.6, 4703.1)	15435.7 (13055.9, 18175.7)	0.8 (0.6, 1.0)	44.3 (32.1, 58.2)	1530.3 (1028.3, 2133.9)	1574.6 (1074.3, 2178.3)
Andean Latin America	4101.4 (3083.1, 5328.4)	18870.2 (15852.6, 22184.3)	0.8 (0.6, 1.1)	47.0 (34.2, 61.9)	1913.9 (1293.3, 2661.6)	1960.9 (1340.8, 2709.7)
Caribbean	4366.2 (3270.5, 5673.5)	17327.2 (14539.7, 20390.9)	2.3 (1.6, 3.3)	126.5 (91.1, 188.7)	1758.7 (1178.4, 2450.9)	1885.2 (1310.5, 2581.7)
Southern Latin America	6863.6 (5029.4, 9026.7)	29007.3 (24332.1, 34014.8)	0.9 (0.8, 1.1)	51.5 (45.5, 58.1)	3141.8 (2127.1, 4371.2)	3193.2 (2177.9, 4422.3)
Tropical Latin America	6142.2 (8107.2, 4524.9,)	24138.4 (20236.9, 28602.5)	1.6 (1.5, 1.8)	91.1 (81.5, 98.7)	2568.7 (1730.2, 3587.4)	2659.8 (1820.9, 3679.2)
Central Asia	5348.1 (3968.5, 7039.8)	16903.1 (13817.6, 20446.3)	0.4 (0.3, 0.4)	19.6 (16.6, 22.8)	1797.4 (1180.7, 2562.5)	1817.0 (1200.8, 2582.7)
Central Europe	7064.6 (5195.5, 9310.6)	21529.9 (17512.9, 26169.9)	0.3 (0.2, 0.3)	14.0 (12.4, 15.7)	2375.0 (1563.5, 3386.2)	2389.0 (1577.4, 3400.4)
Eastern Europe	6392.9 (4738.4, 8429.7)	19266.5 (15719.0, 23362.2)	0.6 (0.5, 0.7)	33.7 (29.6, 38.3)	2072.9 (1366.8, 2955.3)	2106.6 (1400.4, 2989.7)
Central Latin America	5736.4 (4264.6, 7513.1)	24207.3 (20376.0, 28412.4)	2.3 (1.8, 2.7)	129.4 (97.9, 151.6)	2588.2 (1745.4, 3601.4)	2717.6 (1873.7, 3733.9)
Australia	7772.8 (5677.9, 10345.6)	26758.8 (22177.0, 31989.5)	0.5 (0.4, 0.5)	24.5 (21.5, 27.8)	2914.6 (1946.5, 4123.3)	2939.1 (1970.8, 4147.3)
High-income Asia Pacific	6835.5 (5052.1, 8948.3)	26430.1 (22225.2, 31094.4)	0.4 (0.3, 0.5)	21.4 (18.3, 26.0)	2883.4 (1940.4, 4027.7)	2904.9 (1960.9, 4049.1)
High-income North America	6630.9 (5243.9, 8201.0)	29948.6 (27167.5, 32928.8)	0.8 (0.8, 0.9)	44.9 (40.9, 47.7)	3160.0 (2220.7, 4226.6)	3204.9 (2265.0, 4271.3)
Western Europe	5684.8 (4202.0, 7472.6)	21057.8 (17578.3, 24883.5)	0.3 (0.3, 0.3)	14.0 (13.3, 14.7)	2237.0 (1502.1, 3119.5)	2251.0 (1515.9, 3133.4)

MSK: musculoskeletal; YLLs: years of life lost; YLDs: years lived with disability; DALYs: disability-adjusted life years.

Data in parentheses are 95% uncertainty intervals (95% UI).

**Figure 1.**
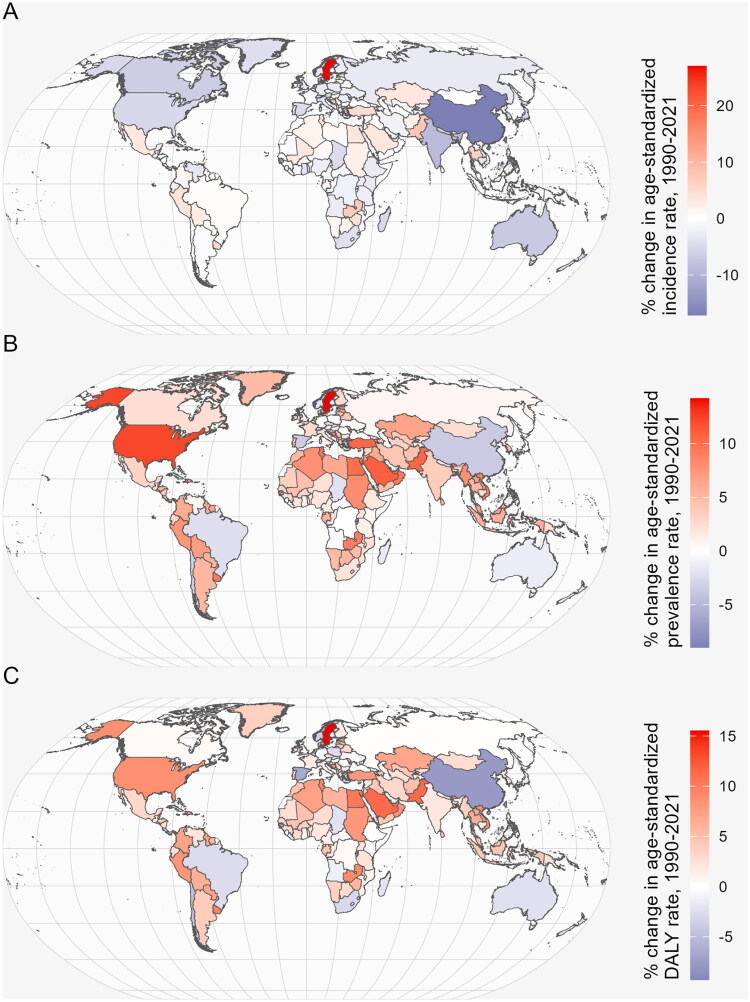
The percentage change of age-standardised incidence (a), prevalence (B), and DALYs rates (C) per 100,000 population of MSK disorders in women of childbearing age at the national level changing trends from 1990 to 2021. DALY: disability-adjusted life years; MSK: musculoskeletal.

From 1990 to 2021, trends in the burden of disease varied significantly across the 204 countries. A total of 99 countries experienced an increase in age-standardized incidence rate (Figure 1(A)), 183 countries and 166 countries showed an upward trend in prevalence (Figure 1(B)) and DALYs rates (Figure 1(C)). Sweden showed notable increases in age-standardized incidence (27.0% [95% confisdence interval (CI) 20.7, 32.7]), prevalence (14.2% [12.6, 15.4]), and DALYs rates (15.5% [13.2, 17.6]). In contrast, China exhibited significant decreases in age-standardized incidence (−17.1% [95% CI −15.3, −17.8]), prevalence (−3.7% [−2.4, −4.8]), and DALYs rates (−7.7% [−6.4, −8.4]) (Figure 1).

**Figure 2.**
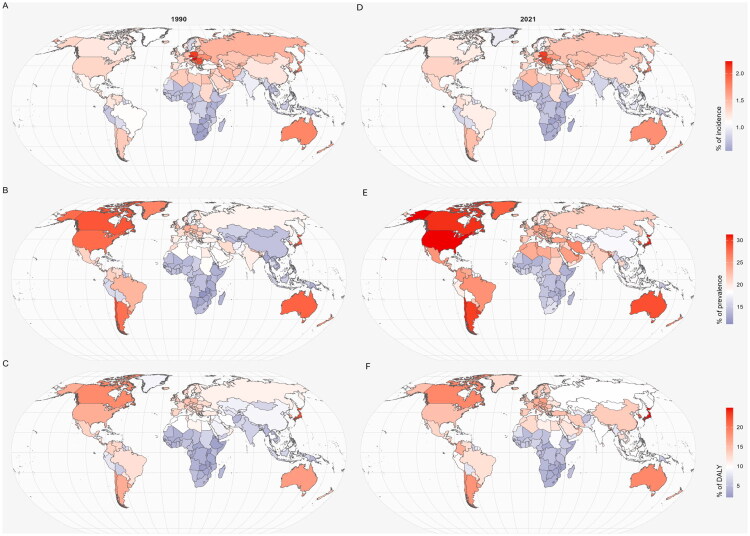
Percentage of age-standardised incidence, prevalence, and DALYs rates relative to MSK disorders in all-cause age-standardised incidence, prevalence, and DALYs rates among women of childbearing age in 1990 (A, B, C) and 2021 (D, E, F). DALY: disability-adjusted life years; MSK: musculoskeletal.

At the national level, the percentage of age-standardized incidence, prevalence, and DALYs rates of MSK disorders in WCBA relative to the age-standardized all-cause incidence, prevalence, and DALYs rates in this population showed different changes in 1990 and 2021. Compared to 1990, in 2021, the percentages of incidence, prevalence, and DALYs rates of MSK disorders in WCBA had risen in 104, 193, and 179 countries, respectively, relative to all-cause rates (Figure 2). In 1990, Hungary (2.2% [95% UI 1.9, 2.6]) had the highest percentage of MSK incidence among WCBA relative to the all-cause incidence rate. By 2021, this distinction shifted to Czechia (2.1% [95% UI 1.8, 2.5]) (Figures 2(A and D)). Regarding prevalence rate, in 1990, Chile (29.4% [95% UI 26.3, 32.5]) had the highest percentage of MSK prevalence among WCBA relative to the all-cause prevalence rate (Figure 2(B)). By 2021, the United States held this position (31.3% [95% UI 29.8, 32.7]) (Figure 2(E)). In 1990, the countries with the highest percentages of DALYs rate for MSK disorders in WCBA were Japan (22.7% [95% UI 19.5, 26.5]), Canada (18.6% [15.7, 22.1]), and Chile (17.9% [15.1, 20.9]) (Figure 2(C)). By 2021, Japan (25.0% [95% UI 21.4, 29.1]) remained the country with the highest percentages of DALYs rate, followed by Singapore (23.2% [19.5, 27.2]) and South Korea (21.8% [18.5, 25.5]) (Figure 2(F)). The country with the most significant percentage increase in DALYs rate relative to the all-cause DALYs rate for MSK disorders among WCBA was Bhutan, with a rise of 7.4% (95% CI 6.3, 8.7).

**Figure 3.**
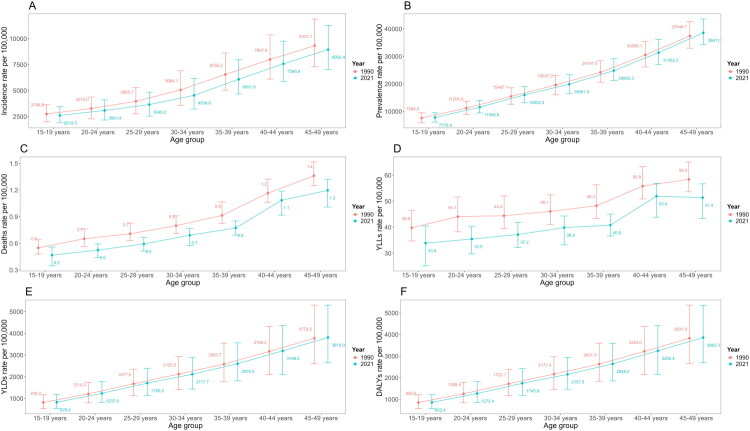
Incidence (a), prevalence (B), deaths (C), YLLs (D), YLDs (E) and DALYs (F) rates per 100 000 population of MSK disorders in women of childbearing age across different age groups in 1990 and 2021. YLLs: years of life lost; YLDs: years lived with disability; DALY: disability-adjusted life years; MSK: musculoskeletal.

In both 1990 and 2021, at the global level, the rates of incidence, prevalence, deaths, YLLs, YLDs, and DALYs per 100,000 population for WCBA increased with age across all age groups (Figure 3), except for a decrease in YLLs among the 45–49 age group in 2021 (Figure 3(D)). Compared to 1990, the incidence, deaths, and YLLs rates for the same age groups decreased in 2021 (Figure 3(A, C, and D)). However, prevalence, YLDs, and DALYs rates increased in 2021, except for a decrease in YLDs rate (from 2125.3 [95% UI 2928.3, 1404.8] to 2117.7 [2890.5, 1437.3]) and DALYs rate (from 2171.4 [2973.7, 1450.2] to 2157.5 [2932.7, 1475.2]) for the 30-34 age group (Figure 3(B, E, and F)).

**Figure 4.**
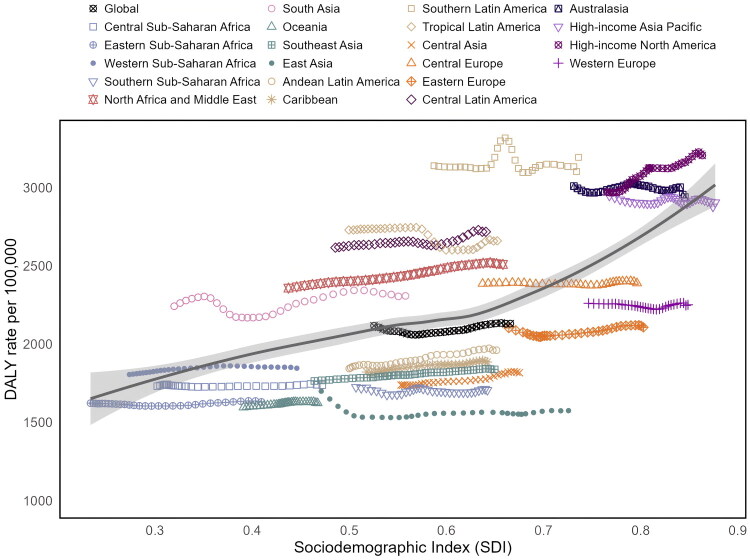
The associations between the SDI and age-standardised DALYs rate per 100,000 population of MSK disorders in women of childbearing age across 21 GBD regions from 1990 to 2021. The solid gray line represents the expected values based on the SDI and DALYs rate across all locations. SDI: Socio-Demographic Index; DALY: disability-adjusted life years; MSK: musculoskeletal; GBD: Global Burden of Disease.

Between 1990 and 2021, there was a positive association between the SDI and age-standardized DALYs rate 100,000 population for MSK disorders in WCBA, both globally and regionally. This indicates that as SDI increased, the burden of MSK disorders also intensified (Figure 4). Over the 32-year observation period, it was found that although the global DALYs rate per 100,000 population for MSK disorders in WCBA has been increasing, it remained slightly below the expected values (below the solid gray line). At the regional level, some regions exhibited significantly higher than expected DALYs rates, including South Asia, North Africa and the Middle East, Central Latin America, Tropical Latin America, Southern Latin America, Australasia, and High-income North America. Conversely, regions such as Eastern Sub-Saharan Africa, Central Sub-Saharan Africa, Oceania, East Asia, Southern Sub-Saharan Africa, Central Asia, Southeast Asia, the Caribbean, Andean Latin America, Eastern Europe, and Western Europe showed that DALYs rates were lower than the expected values (Figure 4).

**Figure 5.**
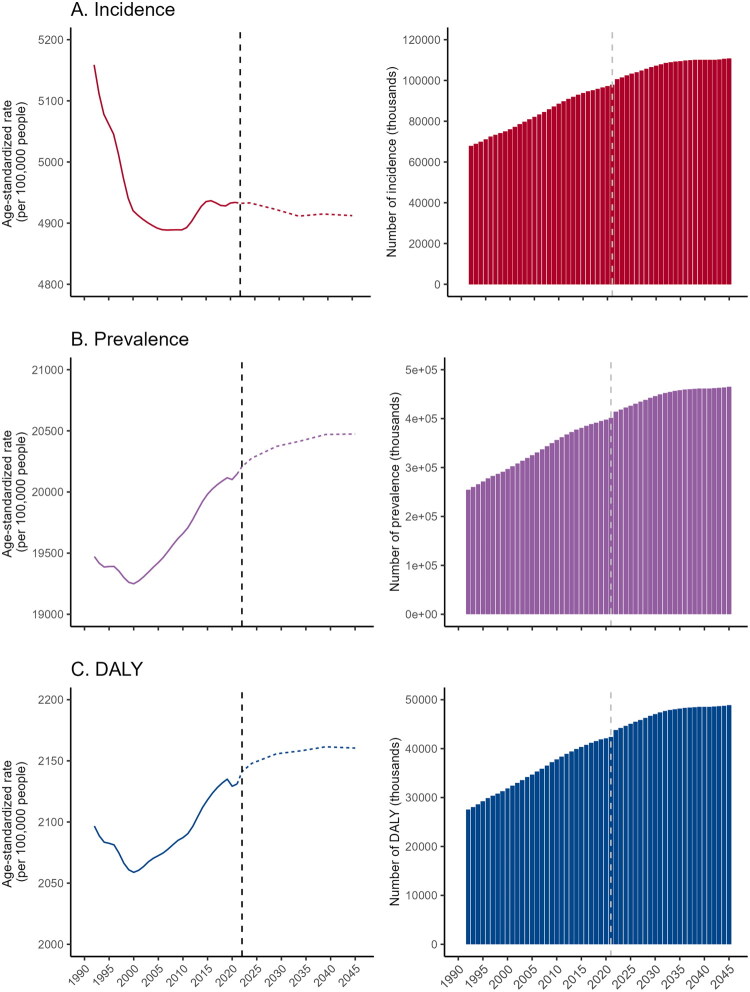
Projection of MSK disorders in women of childbearing age globally from 2022 to 2045. The predicted age-standardised incidence rate and case number (a). The predicted age-standardised prevalence rate and case number (B). The predicted age-standardised prevalence rate and case number (C). MSK: musculoskeletal; DALY: disability-adjusted life years.

Based on projected population changes, the incidence rate of MSK disorders in WCBA was expected to show a slow decline from 2022 to 2045 (Figure 5(A)). Conversely, the prevalence and DALYs rates were anticipated to continue rising during this period (Figure 5(B and C)). By 2045, the age-standardized number of WCBA with MSK disorders was projected to reach 464.9 million, with an age-standardized prevalence rate expected to be 20,474.4 per 100,000 population (Figure 5(B)). The age-standardized number of DALYs was expected to reach 48.8 million, with an age-standardized DALYs rate of 2160 per 100,000 population (Figure 5(C)).

## Discussion

This study provides updated global estimates of incidence, prevalence, and DALYs rates for MSK disorders in WCBA, and for the first time, offers predictive estimates of the burden of MSK disorders in this population through 2045. Our findings highlight a significant global burden of MSK disorders in WCBA. Despite a decline in the incidence rate in some countries, nearly half of the countries and regions worldwide continued to experienced an upward trend from 1990 to 2021. The prevalence and DALYs rates were increasing in most countries and regions. Even in countries where the incidence rate has decreased, there remains significant room for improvement, as the prevalence and DALYs have continued to rise alarmingly. This burden exhibited marked regional disparities and showed a positive association with socio-demographic development.

The burden of MSK disorders in WCBA remained substantial, as evidenced by the global estimates for 2021. Notable disparities of the age-standardized incidence, prevalence, deaths, YLLs, YLDs, and DALYs rates were observed across the 21 GBD regions, reflecting substantial heterogeneity. These findings suggest that similar to the overall burden of MSK disorders [19–22], the burden of MSK diseases in WCBA was not uniformly distributed across different regions, and was influenced by regional socio-demographic factors. The higher prevalence and DALYs rate in High-income North America could be attributed not only to advanced diagnostic capabilities and greater healthcare access but also to differences in awareness and healthcare services. Cultural variations in how conditions like low back pain are perceived and managed may also play a significant role, leading to more cases being reported and managed over longer periods compared to other regions [23]. On the other hand, the high incidence in Australasia might reflect lifestyle factors or occupational risks prevalent in this region [24,25]. The age-standardized incidence, prevalence, and DALYs rates of MSK disorders among WCBA vary significantly across different regions, which may be attributed to the heterogeneity in the interaction between environmental factors and MSK conditions [26–29]. Therefore, flexible health policies tailored to the unique circumstances of each region should be developed to address this complexity effectively.

The significant variability in the burden of MSK disorders in WCBA across countries from 1990 to 2021 highlights the need for nuanced public health strategies. While countries like Sweden experienced significant increases in incidence, prevalence, and DALYs rates – likely due to improved disease management programs and heightened awareness – others, such as China, saw substantial decreases, potentially reflecting effective health policies and socioeconomic advancements [30]. Over the past decade, China’s healthcare reforms have provided equitable access to basic medical services for all citizens, along with reasonable quality assurance and financial risk protection. These reforms have led to substantial progress in disease prevention and control, which may offer valuable insights for global health efforts. In high-burden countries, strengthening prevention programs, enhancing early detection, and ensuring access to effective treatment and rehabilitation are essential [31,32]. These public health efforts are beneficial in reducing the incidence and long-term impact of MSK disorders in WCBA.

Despite stable incidence rates, the increasing prevalence and DALYs highlight the chronic disabling nature. Especially for OMSKD that are more prevalent in WCBA and have a high disability rate, such as systemic lupus erythematosus and systemic connective tissue disorders [33–35]. With improvements in diagnostic and treatment technologies, mortality rates for these diseases have decreased. However, the high disability rates persist, significantly impacting patients’ quality of life as well as their reproductive health and prospects for healthy aging.

To mitigate the growing MSK burden among WCBA, public health efforts should focus on enhancing early intervention and chronic disease management. Given that MSK disorders are shaped by both modifiable factors such as sedentary lifestyle and occupational hazards, and non-modifiable factors such as genetic predisposition and aging, we propose a stratified intervention framework. For modifiable risks, interventions should include ergonomic improvements, digital behavioral programs, and context-specific diagnostic tools. For non-modifiable risks, health strategies should integrate chronic pain management and reproductive services, including joint assessments during preconception counseling and fertility preservation for women with autoimmune diseases. Region-specific resource allocation can further enhance care, with AI-assisted risk prediction in high-SDI areas and mobile clinics in low-SDI regions. These strategies can reduce long-term disabilities, improve quality of life, and help manage the rising prevalence and DALYs rates [36,37].

As with previous findings [38], the global trends in MSK disorders among WCBA in 1990 and 2021 reveal age-dependent increases in incidence, prevalence, deaths, YLLs, YLDs, and DALYs rates across most age groups. Compared to 1990, death rates within the same age groups decreased in 2021, indicating some success in reducing the immediate fatal impacts of MSK disorders. However, the increase in DALYs rates with advancing age observed in 2021 highlights the chronic and disabling nature of MSK disorders, which remain a significant public health challenge. Early intervention is therefore crucial for reducing DALYs rate in later stages and improving outcomes, particularly in younger age groups.

This study reveals a complex relationship between SDI and DALYs rate for MSK disorders in WCBA. As with previous findings [39], while higher SDI is generally associated with increased DALYs rate, significant regional differences persist. The observed variability suggests that factors beyond SDI, such as regional health policies and healthcare infrastructure, also play a key role in shaping the burden of MSK disorders. This underscores the need for targeted health interventions that consider both sociodemographic development and regional health disparities.

Our projections to 2045 suggest a continued rise in the prevalence and burden of MSK disorders among WCBA, despite a declining incidence rate. First, the future growth of the population and the early onset of MSK diseases may exacerbate the disease burden [40]. Second, the burden of MSK diseases during the reproductive age may increase in low SDI regions due to a lack of comprehensive preventive and therapeutic measures, and the existing disease burden in these areas may be underestimated because of inadequate medical information collection systems. Finally, current interventions can only alleviate the disease burden and achieve clinical remission as much as possible, but relapses are common. For example, immunosuppressants, biological agents, stem cell, CAR-T therapies, corticosteroids, and non-steroidal anti-inflammatory drugs are used to control diseases with a heavy burden such as systemic lupus erythematosus, rheumatoid arthritis, systemic connective tissue disorders, and inflammatory polyarthropathies [41,42]. This underscores the importance of ongoing public health efforts to address the growing burden of MSK disorders, particularly in high-risk regions and populations. Effective interventions, tailored to regional needs and resources, are essential to mitigate the impact of these conditions on women’s health and quality of life globally.

This study has several limitations that must be acknowledged. First, the inability to assess each individual disease within the MSK disorder category, such as the 15 high-burden diseases included in OMSKD, may lead to an underestimation of the disease burden for specific conditions. The high prevalence of these diseases suggests substantial overlap and potential dependency comorbidities among various MSK disorders. This overlap indicates that our current burden estimates might be underestimated rather than overestimated. Therefore, there is an urgent need for further population-based data collection and the delineation of disease subtypes within the MSK disorder category to better inform health policies addressing these high-prevalence conditions. Second, the inherent uncertainties in the GBD methodology were not considered. Data scarcity, particularly in developing countries, poses another challenge. As with other studies [43,44], estimates for data-deficient regions are primarily generated using the DISMOD-MR 2.1 model, resulting in large uncertainty intervals. Additionally, using insurance claims data from the United States and Poland could contribute to underestimation, as not all individuals classified with MSK disorders seek healthcare, particularly those with chronic symptoms. Finally, the lag in GBD data may affect the timeliness and relevance of the findings.

In conclusion, the burden of MSK disorders among WCBA is already substantial and is expected to increase further in the future. Despite the observed decline in age-standardized incidence rate of MSK disorders among WCBA in half of the regions and countries globally, the age-standardized prevalence and DALYs rates have shown an adverse increasing trend. This indicates that the chronic and disabling impact of MSK disorders on this population is intensifying, stratified and targeted healthcare strategies are essential to improve early diagnosis and treatment. The health policies of countries like China, where the DALYs rate has decreased, may offer valuable insights for global prevention efforts.

## Supplementary Material

Supplementary Table.docx

## Data Availability

The datasets supporting the conclusions of this study can be downloaded from the official website of the GBD database (https://vizhub.healthdata.org/gbd-results/) and are also available from the corresponding author upon reasonable request.
