# Supplementary material for: Musculoskeletal disorders in women of childbearing age: global trends, socio-demographic disparities, and future projections
Source: Ann Med. 2025 Jul 20;57(1):2532860. doi: 10.1080/07853890.2025.2532860 (PMC12278454; doi:10.1080/07853890.2025.2532860)
Supplement: Supplementary Table.docx [file IANN_A_2532860_SM0347.docx]

**Supplementary Table 1** definitions and corresponding ICD codes for musculoskeletal disorders.

| **Cause** | **Definition** | **ICD-10** | **ICD-9** |
| --- | --- | --- | --- |
| Musculoskeletal disorders | Six major categories, including rheumatoid arthritis, osteoarthritis, low back pain, neck pain, gout, and other musculoskeletal disorders. | L93, M00–M02, M05–M06.9, M08.0–M08.8, M08, M10, M11–M13, M16, M17, M18, M19, M20–M25, M30–M35, M40–M43, M45–M46, M54.2, M54.3, M54.4, M54.5, M60–M63, M65–M68, M70–M73, M75–M79, M80–M85, M86, M87–M90, M91–M94, M95–M99 | 274, 710.0, 711, 712–713, 714–714.3,  714.8–714.9, 715, 716–719, 710.1–710.9,  737, 720–721, 723.1, 724, 725, 726–728,  729, 733.0–2, 730.1–730.3, 730.7–9, 731,  733.3–9, 732, 734–736, 738–739 |
| Rheumatoid arthritis | An autoimmune disease primarily characterized by joint swelling, pain, and deformity, accompanied by systemic symptoms.The GBD database adopts the 1987 ACR classification criteria. | M05–M06.9, M08.0–M08.8 | 714–714.3, 714.8–714.9 |
| Osteoarthritis | Used involves symptomatic Osteoarthritis that has been radiologically confirmed as Kellgren-Lawrence grade 2 to 4. | M16, M17, M18, M19 | 715 |
| Low back pain | Pain in the lower back (the posterior region extending from the lower edge of the 12th rib to the gluteal folds) with or without localized pain in one or both lower limbs, lasting for at least one day. | M54.3, M54.4, M54.5 | 724 |
| Neck pain | Pain in the neck, with or without localized pain into the upper limbs, lasting for at least one day. | M54.2 | 723.1 |
| Gout | A rheumatic disease characterized by non-infectious acute arthritis due to the deposition of urate crystals.The GBD adopts the case definition for gout provided by the American College of Rheumatology in 1977. | M10 | 274 |
| Other musculoskeletal  disorders. | A heterogeneous residual category encompassing a broad range of muscle, bone, and ligament disorders not covered by the five MSK conditions(rheumatoid arthritis, osteoarthritis, low back and neck pain, and gout) defined by GBD.These include 15 disease categories: lupus erythematosus, infectious arthropathies, inflammatory polyarthropathies, other joint disorders, systemic connective tissue disorders, deforming dorsopathies, spondylopathies, muscle disorders, disorders of the synovium and tendon, other soft tissue disorders, disorders of bone density and structure, osteomyelitis, other osteopathies, chondropathies, and other disorders of the musculoskeletal system and connective tissue. | L93, M00–M02, M08, M11–M13, M20–M25,  M30–M35, M40–M43, M45–M46, M60–M63,  M65–M68, M70–M73, M75–M79, M80–M85,  M86, M87–M90, M91–M94, M95–M99 | 710.0, 711, 712–713, 716–719, 710.1–710.9,  737, 720–721, 725, 726–728, 729, 733.0–2,  730.1–730.3, 730.7–9, 731, 733.3–9, 732,  734–736, 738–739 |

GBD=Global Burden of Disease.
